# Polμ Deficiency Increases Resistance to Oxidative Damage and Delays Liver Aging

**DOI:** 10.1371/journal.pone.0093074

**Published:** 2014-04-01

**Authors:** Beatriz Escudero, Daniel Lucas, Carmen Albo, Suveera Dhup, Jeff W. Bacher, Aránzazu Sánchez-Muñoz, Margarita Fernández, José Rivera-Torres, Rosa M. Carmona, Encarnación Fuster, Candelas Carreiro, Raquel Bernad, Manuel A. González, Vicente Andrés, Luis Blanco, Enrique Roche, Isabel Fabregat, Enrique Samper, Antonio Bernad

**Affiliations:** 1 Departamento de Cardiología Regenerativa, Centro Nacional de Investigaciones Cardiovasculares (CNIC), Madrid, Spain; 2 Departamento de Inmunología y Oncología, Centro Nacional de Biotecnología/CSIC, Campus Universidad Autónoma de Madrid, Madrid, Spain; 3 Genetic Analysis Group, Promega Corporation, Madison,Wisconsin, United States of America; 4 Departamento de Bioquímica y Biología Molecular II, Universidad Complutense, Madrid, Spain; 5 Departamento de Epidemiología, Aterotrombosis e Imagen, Centro Nacional de Investigaciones Cardiovasculares, Madrid, Spain; 6 Institute of Bioengineering, Miguel Hernandez University, Elche (Alicante), Spain; 7 Centro de Biología Molecular Severo Ochoa/CSIC, Cantoblanco, Madrid, Spain; 8 CIBERobn(CB12/03/30038) Instituto de Salud Carlos, Madrid, Spain; 9 Bellvitge Biomedical Research Institut (IDIBELL), L'Hospitalet de Llobregat, Barcelona, Spain; National Institute of Environmental Health Sciences, United States of America

## Abstract

Polμ is an error-prone PolX polymerase that contributes to classical NHEJ DNA repair. Mice lacking Polμ (Polμ^−/−^) show altered hematopoiesis homeostasis and DSB repair and a more pronounced nucleolytic resection of some V(D)J junctions. We previously showed that Polμ^−/−^ mice have increased learning capacity at old ages, suggesting delayed brain aging. Here we investigated the effect of Polμ^−/−^ deficiency on liver aging. We found that old Polμ^−/−^ mice (>20 month) have greater liver regenerative capacity compared with wt animals. Old Polμ^−/−^ liver showed reduced genomic instability and increased apoptosis resistance. However, Polμ^−/−^ mice did not show an extended life span and other organs (e.g., heart) aged normally. Our results suggest that Polμ deficiency activates transcriptional networks that reduce constitutive apoptosis, leading to enhanced liver repair at old age.

## Introduction

There are many types of DNA lesion, but base modifications, single-strand breaks (SSBs) and double-strand breaks (DSBs) are the most frequent forms, with DSBs being the most harmful (review by [1]). Two main pathways are responsible for DSB repair: Non Homologous End Joining (NHEJ) and Homologous Recombination (HR) (reviewed [2]). NHEJ is a critical mechanism for preventing the adverse effects of DSBs and its deficit is associated with premature aging [3]. Polμ is a widely expressed error-prone enzyme [4], [5] and mice lacking Polμ show a specific and pronounced nucleolytic resection of V(D)J junctions [6]. After TdT, Polμ is the second-most promiscuous PolX polymerase, as it has intrinsic terminal transferase activity and has the unique ability to direct template-dependent synthesis across a DSB with no terminal microhomology [7], [8]. Polμ was recently shown to be required for hematopoiesis homeostasis and DSB repair *in vivo*, and its deficiency promotes genetic instability in primary mouse embryonic fibroblasts (MEFs) and bone marrow (BM) cells [9], [10]. Recently, however, we reported that old Polμ^−/−^ mice have substantially above-normal maintenance of learning abilities and show fewer signs of brain aging [11], an unexpected phenotype for the elimination of a DNA repair function. This phenotype is associated with reduced error-prone DNA oxidative repair activity and a more efficient mitochondrial function [11]. Here we investigated whether altered DNA repair in Polμ^−/−^ mice leads to enhanced organ function in old age and a net delayed aging. Our results demonstrate that, despite reduced DSB repair capacity, Polμ^−/−^ have an enhanced liver regenerative potential during aging, not paralleled in other organs.

## Materials and Methods

### Mice and treatments

The generation of Polμ^−/−^ mice was previously described [5]. Mutant and wild-type (wt) mice were bred in our specific pathogen-free (SPF) facilities and were routinely screened for pathogens. Most experiments were carried out with animals in the original mixed 129sv×BALB/c background. Where indicated, experiments were carried out in the C57BL/6 background. Longevity and fertility of both colonies were monitored continuously, and when indicated weight and food consumption was studied in age-matched groups of animals. Glucose tolerance tests were performed by starving the mice for 16 hours and then injecting glucose (1 g/kg) intraperitoneally. To evaluate resistance to intense oxidative stress, mice (Polμ^−/−^ and wt) were injected (ip; 70 mg/kg) with paraquat (Sigma, St. Louis, MO, USA) and closely monitored for early detection of moribund symptoms. At the onset of moribund symptoms, animals were sacrified and organs extracted for further analysis. For evaluation of the effect of oxidative stress on microsatellite stability, animals were treated with lower doses. All experiments were performed according to Spanish and European regulations for the use and treatment of experimental animals, with the approval of the ethics committees of the Centro Nacional de Biotecnología (CNB) and Fundación Centro Nacional de Investigaciones Cardiovasculares (CNIC).

### Heart analysis and blood pressure

Mice were anesthetized with 2.5% sevoflurane and electrocardiographic parameters were assessed with a Vevo 2100 transthoracic echocardiograph equipped with a 30-MHz ultrasound probe (PR segment and QRS complex); data were analyzed with VevoStrain (Visual Sonic). Blood pressure (systolic and diastolic) and heart rate were measured in trained conscious mice using a noninvasive automated tail-cuff device (Visitech System BP2000, NC). Mice were trained daily over one week and 20 measurements were then taken once a week at the same time in the morning. To increase accuracy, the first 10 measurements were discarded and the mean of the remaining 10 readings were used for analysis.

### Partial hepatectomy

Aged mice (18 to 23 months old) were subjected to a standard 70% hepatectomy [12]. Briefly, mice were anesthetized with a mixture of isoflurane/oxygen, and right medial, left medial, and left lateral lobes were excised by ligation, resulting in removal of 70% of the hepatic mass. At the indicated times post-surgery, livers were harvested and processed for subsequent analysis.

### Cell death analysis

Apoptotic cells in frozen liver-tissue samples (−80°C; O.C.T.-included; 14-months-old mice, n = 3) were detected by TUNEL staining, using the In Situ Death Detection Kit (Roche, 1 684 795). Images were acquired with a Leica DM2500 confocal microscope.

### ROS and oxidative stress analysis

Spleen and thymus were disaggregated in PBS and erythrocytes were lysed as described above. Samples were incubated at 37°C, in the dark, with 5 µM 2′,7′–dichlorofluorescein diacetate (DCFDA), 5 µM dihydroethidium (DHE), 1 µM MitoTracker Deep red 100 nM probes or 5 µM tetramethylrhodamine methyl ester (TMRM) for 30 min at 37°C (all probes from Molecular Probes, Invitrogen). Staining with TOPRO (for cells incubated with DCFDA and DHE) or DAPI (for cells incubated with MitoTracker) were used to distinguish live from dead cells. Cells were analyzed in a FACSCanto II cytometer (Becton Dickinson). MEFs were trypsinized and resuspended in HBSS/Ca/Mg phenol-red-free medium (Sigma-Aldrich) at 10^6^ cells/ml, and processed in the same way. Protein carbonyls and thiobarbituric acid reactive substances (TBARS), as macromolecular oxidative damage, were determined as described in the supporting information.

### Sister chromatid exchange (SCE) assay

Cells were incubated in medium containing 10 µM BrdU for two cell divisions. Colcemid (0.05 µM; Gibco, 15210-040) was added for the last 1.5 h of culture (3.5 h for MEFs). After swelling in hypotonic buffer (0.56% potasium chloride; 25 min at 37°C), cells were fixed in methanol/acetic acid (3∶1). For BrdU detection, metaphases were spread and slides were incubated in 2X SSC with 0.5 µg/mL Hoechst 33258 for 15 min, crosslinked for 15 min in 2X SSC, and dehydrated through an ethanol series. After air drying, slides were blocked in 1%BSA/PBS, denatured in 0.07N NaOH for 2 min, neutralized in PBS pH 8.5 for 5 min, permeabilized in 0.5%Tween-20/1%BSA/PBS for 5 min twice, and incubated with FITC-conjugated anti-BrdU antibody (347583, BD Biosciences, San Jose, CA, USA) diluted 1∶1 in 0.5% Tween-20/3%BSA/PBS. Slides were then washed three times in 0.1%Tween-20/PBS for 5 min, dehydrated, air dried and mounted in Vectashield with DAPI (Vector Laboratories, Burlingame, CA, USA).

### Quantitative real time PCR (RT-PCR)

Total RNA was isolated from liver (14-month-old mice, n = 4) using TRI REAGENT (Sigma, P/N: T9424). cDNA was synthesized using the Reverse Transcription kit (Promega, P/N: A3500). Gene expression was evaluated by TaqMan assay and mRNA levels were normalized to a standard housekeeping gene (beta-actin). Each reaction contained 10 ng cDNA, TaqMan 2X PCR master mix (Applied Biosystems, P/N: KP0054) and 20X Gene TaqMan assay, using the primers indicated in Table S2. Real-time quantitative PCR assay was performed using the Applied Biosystems 7000 Sequence Detecting system. Transcript levels are calculated as 2^−Δct^, where Δct is the *ct* value obtained after normalization to the internal beta-actin expression control.

### Statistical analysis

Data are expressed as mean ± SEM. Statistical differences were analyzed by Student's-test for unpaired samples and normally distributed data sets. * p≤0.05; ** p≤0.01; *** p≤0.001.

## Results

### Polμ^−/−^ mice show enhanced preservation of liver function with age

**Figure 1:**
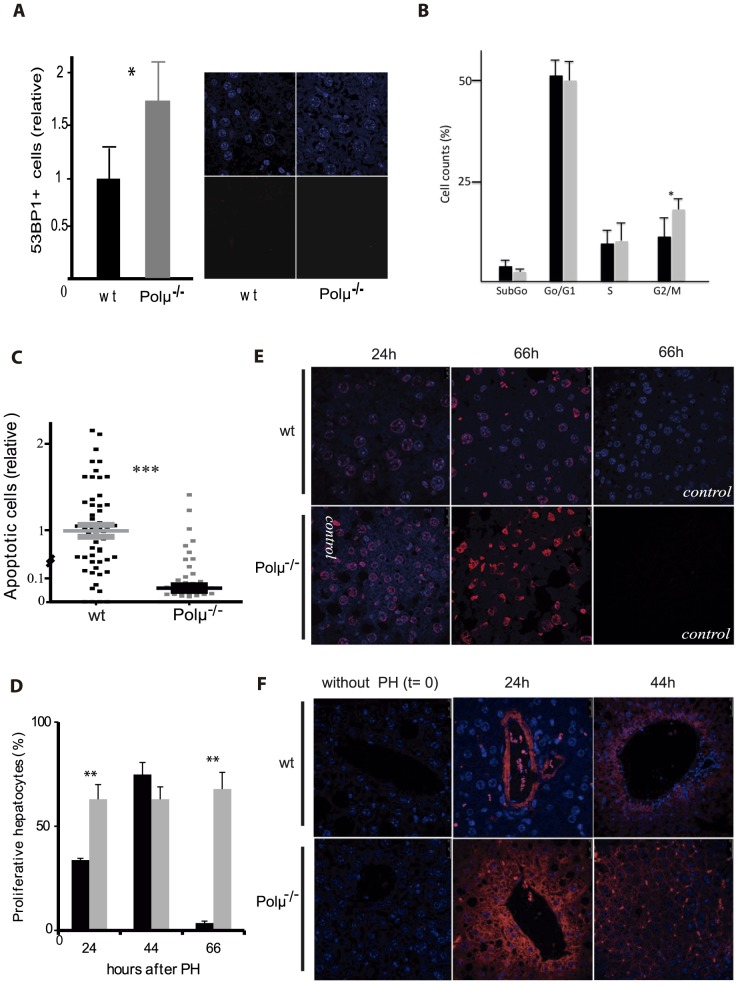
Improved liver function in old Polμ^−/−^ mice. (**A**) Analysis of 53P1-positive cells in liver of old (>20 months) wt (n = 6; black bars) and Polμ^−/−^ mice (n = 6; gray bars). (**B**) Cell cycle analysis of freshly prepared liver suspensions from wt (black bars) and Polμ^−/−^ mice (gray bars), after staining with PI. (**C**) TUNEL assay in cryopreserved liver tissue from adult (14 m) mice. Percentages of positive (apoptotic) cells per field are shown. Three livers per genotype were analyzed (4771 wt cells and 8289 Polμ^−/−^ cells) and individual values (relative to wt) and means ± SEM are shown. (**D**) Percentage of proliferating (PH3-positive) cells in livers of old (18–23 m) wt (black) and Polμ^−/−^ mice (gray) after partial hepatectomy (PH). Percentages of PH3-positive cells are presented as the mean ± SEM of two-three animals per condition; n = 11. Polμ^−/−^ animals survived surgery better than wt animals (82%; 9/11 vs 64%; 7/11). (**E, F**) Representative images of immunostaining for PH3 (E) and CK19 (F) in sections of old (18–23 m) Polμ^−/−^ and wt liver at the indicated times after PH. Control indicates negative controls without the primary antibody.

To investigate whether altered DNA repair in Polμ^−/−^ mice leads to enhanced preservation of organ function and a net delayed aging, we first focused on liver. Staining with anti-53BP1 revealed a 2-fold higher number of unrepaired DSB in hepatocytes from old (18 m) Polμ^−/−^ mice (n = 6) compared with age-matched wt controls (Figure 1A). Polμ deficiency thus delays liver DSB repair as previously demonstrated in hematopoietic cells [9], [10]. Similar results were found by analysis of γ-H2AX foci in liver, bone marrow and thymus, but differences between Polμ^−/−^ and wt mice were not so evident in spleen (Table 1). Cell cycle analysis revealed a significantly higher percentage of G2/M hepatocytes in Polμ^−/−^ mice (20.72±2.91 vs 11.4±4.91 in wt; p = 0.011) (Figure 1B). In addition, TUNEL immunofluorescence revealed a 10-fold lower level of apoptotic cells in 14-month-old Polμ^−/−^ liver (Figure 1C). To investigate whether the downmodulated apoptosis in Polμ^−/−^ liver is associated with a more robust maintenance of organ function, we evaluated liver regeneration in age-matched old mice (18–23 m; n = 22). Mice were subjected to partial hepatectomy (PH) and hepatic regeneration capacity was assessed in survivor animals (n = 16). Polμ^−/−^ animals survived surgery better than wt animals (82%; 9/11 vs 64%; 7/11) and demonstrated a higher regeneration capacity, measured primarily as the level of proliferating (phospho-H3 positive) hepatocytes (Figure 1D, E). At 24 h post-PH, the rate of regeneration in Polμ^−/−^ mice was almost double that in controls. Moreover, the number of proliferating hepatocytes in Polμ^−/−^ mice was 5-fold higher than in controls at 66 h post-PH, indicating that regeneration is maintained for longer in Polμ^−/−^ mice. The post-PH Polμ^−/−^ liver (Figure 1F) also showed stronger expression of cytokeratin 19 (CK19), a marker associated with hepatic progenitors [13]; at 24 h after PH, CK19-positive liver cells were 2-fold more abundant in Polμ^−/−^ liver than in wt (62%±28.76; p = 0.116 vs 30.2%±24.12; p = 0.003). These results indicate that old Polμ^−/−^ liver has enhanced regeneration capacity.

**Table 1:** Summary of different physiological parameters analyzed in old Polμ^−/−^ mice.

	Liver	BM	Heart	Other organs	Global	MEFs	Other
Cell Cycle	x 2(a)						
Apoptosis	x 0.1						
Senescence						**++** (b)	
Mitochondrial Activity	**+/−**			x 1.6 (Br)			
Autophagy	**+/−**		**+/−**	**+/−** (Br)			
8-oxoG bypass	**+/−**			x 0.2 (Br)			
Genetic stability	**+** (c)	x 0.7(d)				x 0.3(d)	
*MSI*	**+/−**						
Telomere Lenght	−	**−**				**+/−**	
DSB repair							
*53BP1 foci*	x 2						
*γ-H2AX foci*	x 1.7	> x 2		x5 (Thy); **+/−** (Sp)			
*Radiation sensitivity*		x 2.5(e)				> x 4.5	x 4(f)
*8-oxoG bypass*	**+/−**			x 0.2 (Br)			
Aging							
*Histology*	−			− (Sk, Ov, Panc)			
*Immunity*				**+** (CD4/CD8; PB)			
*Biochemistry*							
Colesterol				**−**			
Triglycerides				**−**			
Liver function				**+/−** (g)			
Glucose				**−**			
*Glucose tolerance*				**+/−**			
*IGF1, GH*				**+/−**			
P-Hepatect. Recovery(h)	**+**						
Heart function				**+/−**			
ROS	**+/−** (i); **++** (j)	**−**		**−** (Thy); **+/−** (Sp)		**+** (k)	
Paraquat Resistence (l)					**+**		
HR/NHEJ							
*HR (SCE)*		**+/−**		**+/−** (B cells)		**+**	**+** (m)
*HR expression* (n)	**+**	**+**					
Tumor susceptibility(o)				**+/−** (Thy)	**+/−**		
Life span					**+/; +/−** (p)		

Numbers indicates the ratio KO/WT for each parameter. Footnotes.

(a) Corresponding specifically to G2/M cells;

(b) Evaluation of senescence *ex vivo*, at atmospheric concentration of O_2_;

(c) Ploidy (≥4n) evaluation after PI staining;

(d) Aneuploidy and translocation frequency evaluation;

(e) Evaluated on CFU-GM progenitors;

(f) Evaluated in cell lines;

(g) ALT/GPT; AST/GOT; Billirubin and Albumin;

(h) P-Hepatect. Recovery: partial hepatectomy recovery;

(i) Protein carbonyls;

(j) peroxided lipids;

(k) Values obtained at atmospheric oxygen;

(l) Paraquat Resistance, (70 mg/kg; ip);

(m) Analysis of CHO-DN cell line, at atmospheric oxygen;

(n) HR express. qRT-PCR analysis of selected functions involved in HR;

(o) Tumor susceptibility. Evaluation of spontaneous tumor incidence and susceptibility to thymic lymphomas by low dose of radiation;

(p) Evaluations in 129/BALBc and B6 backgrounds, respectively; Abbreviations: Thy, thymus; Sp, spleen; Br, brain, Sk, skin; Ov, ovary; Panc, Pancreas; PB, peripheral blood. Symbols; (**++**), (**+**), (**+/−**) and (−) indicates that values for KO animals were clearly higher, superior, similar and reduced, respectivelly, in comparison with control (wt) animals.

**Figure 2:**
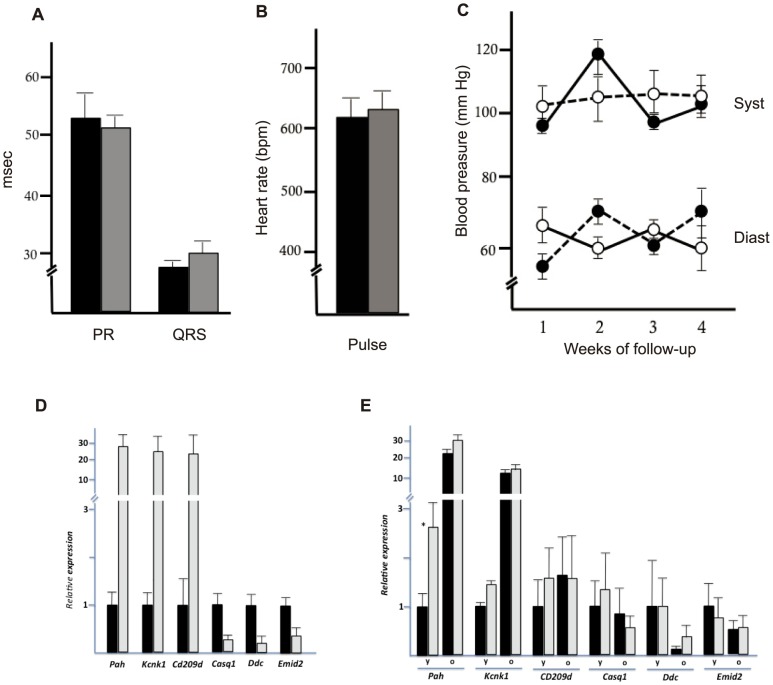
Heart molecular signature and cardiovascular phenotype of Polμ^−/−^ mice. (**A–C**) Cardiovascular parameters in old Polμ^−/−^ (gray) and wt (black) mice (16–18 m). (**A**) Electrocardiographic parameters; QRS complex and PR segment intervals are shown in miliseconds (msec). (**B**) Heart rate (beats per minute; bpm). Data are means ±SD of weekly readings collected over a 4-week period. (**C**) Systolic and diastolic blood pressure (mm Hg) in wt (black) and Polμ^−/−^ mice (white) over a 4-week period. Results are means ± SD (n = 3 mice). (**D**) After DNA array expression analysis of hearts from old (20–24 m) and young (3–4 m) wt mice, six genes showing an aging-related profile were selected and confirmed by qRT-PCR. Data from old mice (black) are presented relative the expression level of each gene in young mice (gray). (**E**) Comparative gene expression analysis in young (y; 8–12 weeks) and old (o; 20–24 m) heart tissue from wt (black) and Polμ^−/−^ mice (gray). Data were normalized to the expression level of each gene in young wt mice. Results, expressed as 2∧–(DCT), are mean values ± SD (n = 3).

Electrophysiological studies in old (18–23 months; n = 5) wt and Polμ^−/−^ animals revealed no alterations in heart function of Polμ^−/−^ mice compared with wt mice (Figure 2A–C), and monitoring over 4 consecutive weeks revealed no significant differences in PR segment & QRS complex (Figure 2A), heart rate (Figure 2B), or systolic and diastolic blood pressure (Figure 2C). To confirm the absence of delayed aging in the hearts of old Polμ^−/−^ mice we conducted a direct molecular evaluation; by DNA array expression analysis we defined a panel of 6 genes that are modulated during aging in several mouse strains. In wt mice, *Pah*, *Knck1* and *Cd209d* were upregulated during heart aging, whereas *Casq1, Ddc and Emid2* were downregulated (Figure 2D). Comparison of age-related alterations to these gene functions in young vs old wt and Polμ^/−^ hearts revealed no significant differences (Figure 2E), confirming that Polμ deficiency does not affect heart function.

In contrast, old Polμ^−/−^ animals (18–24 months) showed better preservation of subcutaneous adipose tissue (Figure S1A), compatible with delayed aging [14]; histological analysis confirmed a similar situation in ovary and pancreas (Figure S1B). Old Polμ^−/−^ mice also maintained peripheral blood populations of CD4+ and CD8+ cells at higher levels than wt counterparts (Figure S1C), and had lower fasting serum levels of cholesterol, triglycerides and glucose (Figure S1D). Given the correlation between high-serum glucose, triglycerides and cholesterol with aging [15] these results suggest a plausible association with the improved functional preservation phenotype observed in some tissues of Polμ^−/−^ mice. Female wt and Polμ^−/−^ mice had identical rates of spontaneous tumor formation (Figure S1E), and identical survival curves after induction of thymic lymphomas with low dose γ-irradiation (Figure S1F).

Although most of the phenotypic alterations in Polμ^−/−^ mice were reproduced in the 129xBALB/c hybrid and C57BL/6 genetic backgrounds, lifespan analysis did not yield conclusive results. Polμ^−/−^ mice in the 129xBALB/c hybrid background, under pathogen-free husbandry conditions, live longer than their wt littermates, housed in parallel (Figure S2A). However, this difference was not confirmed in the C57BL/6 background (Figure S2B) and we therefore cannot conclude any longevity effect directly related to elimination of Polμ.

### Polμ deficient liver shows enhanced genomic stability

**Figure 3:**
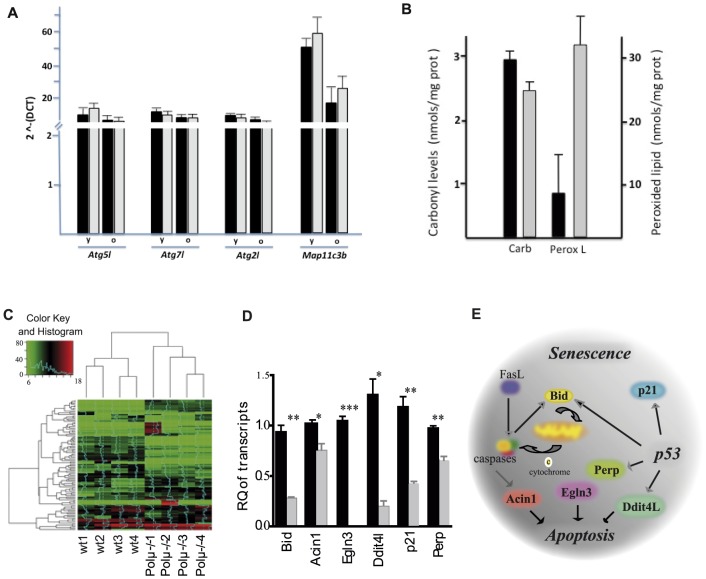
Liver from old Polμ^−/−^ mice shows fewer aging-related features than liver from age-matched wt controls. (**A**) Involvement of autophagy activity in the Polμ^−/−^ lifespan extension phenotype. Heart samples from old (18–25 m; o) and young (8–12 w; y) Polμ^−/−^ (gray bars) and wt mice (black bars) were analyzed by qRT-PCR for the expression of a panel of genes essential for autophagy (Atg2l, Atg5l, Atg7l and Map11c3b). Results, expressed as 2∧–(DCT), show mean values ± SD (n = 5). (**B**) Protein carbonyls (Carb) and peroxided lipids (PeroxL; thiobarbituric acid reactive substances, TBARS), as macromolecular oxidative damage, were determined (nmol/mg prot), as described in the supporting information, in perfused liver of old (>20 months) Polμ^−/−^ (black bars) and wt gray bars) mice Results are mean values ± SD (n = 3). (**C**) Heat map of microarray expression data for livers from adult (14 m) wt and Polμ^−/−^ mice. The relative gene expression scale is shown, with normalized scores ranging from 0 to 20. Gene cluster and case cluster dendrograms are plotted to the top and left. (**D**) Microarray data validation. mRNA levels were determined by TaqMan RT-PCR in livers from adult wt (black) and Polμ^−/−^ mice (gray). Each determination was performed in triplicate and normalized to β-actin expression. Data are the means ± SEM of 4 samples per genotype, represented relative to expression in age-matched wt liver. (**E**) Scheme of the interaction network among apoptosis-related or p53-inducible genes downregulated in old Polμ^−/−^ liver.

To investigate the mechanisms through which Polμ deficiency preserves liver function with age, we first analyzed biochemical parameters. Serum levels of IGF-1 and growth hormone (GH) at 11–25 months of age did not show significant differences in Polμ^−/−^ vs wt mice (Figure S3A; unpublished data). In addition analysis in adult-old (11–25 months) mice of serum parameters related with liver function (ALT/SGPT; AST/SGOT; bilirubin and albumin) did not reveal significant difference (unpublished data). Modest differences were found in glucose tolerance tests in 3-month-old Polμ^−/−^ mice, but these were not maintained in 8-month-old animals (Figure S3B). Altered Insulin/IGF-like signalling (IIS) therefore does not seem to play an important role in the functional preservation phenotype of Polμ^−/−^ mice liver. Another main contributor to function preservation is telomere maintenance (reviewed by [16]); however, we found no positive effect of Polμ deficiency on telomere length, either in primary Polμ^−/−^ MEFs (Figure S3C) or in BM or hepatocytes (unpublished data). Autophagy, another process typically modified with age, was also not significantly altered in Polμ^−/−^ liver (Figure 3A) or brain [11]. Oxidative damage is also an important contributory factor in aging [17]. Comparative analysis of peroxides levels (DCFDA staining) in BM and thymus revealed no genotype-related differences in young adult animals but a significant reduction of peroxides in aged Polμ^−/−^ mice (>19 months) compared with wt (Fig. S3D,E). Analysis of thymus and BM (unpublished data) with specific reagents for different ROS species demonstrated that Polμ^−/−^ and wt cells have similar ROS levels in young animals, whereas cells from old Polμ^−/−^ mice present significant differences from aged-matched controls for all probes used, affecting most free-radicals analyzed: DCFDA (H_2_O_2_, HO^.^, ROO^.^), DHE (O_2_^−^) and MTG and TMRM (mitochondrial permeability and energetic state) (Fig. S3F). ROS levels in MEFs grown at different oxygen concentrations demonstrated that culture at high oxygen concentration (20%) consistently induced higher ROS levels in Polμ^−/−^ MEFs than in wt MEFs, and these differences were almost eliminated when the cultures were maintained at low (5%) oxygen (Fig. S3G). This result is in agreement with the proneness of Polμ^−/−^MEFs to senescence at high oxygen concentrations [9], [10] and indicates a strong dependence of Polμ^−/−^ phenotype on oxidative stress levels. Comparative analysis of macromolecular oxidative damage in old (>20 months) Polμ^−/−^ mice rendered disparate results (Figure 3B); whereas the protein carbonyl level is similar to wt animals, the representation of peroxided lipids (thiobarbituric acid reactive substances; TBARS) is clearly overrepresented in Polμ^−/−^ mice.

We next analyzed gene expression in total liver from 14-month-old wt or Polμ^−/−^ mice. This analysis detected 1556 differentially expressed genes (Figure 3C) and 49 potentially deregulated cellular processes (Table S1; Gene Ontology). Preliminary analysis established a significant alteration in liver metabolism but did not clearly implicate alterations related to caloric-restriction programs [18] or to similarity to alterations reported in brain [11]. We concentrated our analysis on apoptosis mediators and genes regulated by p53, processes typically upregulated with aging, in conjunction with immune, stress and defense responses [19]. qRT-PCR validation confirmed a set of genes significantly (p<0.05) downregulated in Polμ^−/−^ livers that included *Bid, P21, Perp, Acin1, Egln3* (*Pdh3*) and *Ddit4l/Redd2* (Figure 3D), all of which can be modeled in a minimal network related to apoptotic processes (Figure 3E). These results suggest that Polμ deficiency promotes a transcriptional program that promotes apoptosis resistance in the old liver.

**Figure 4:**
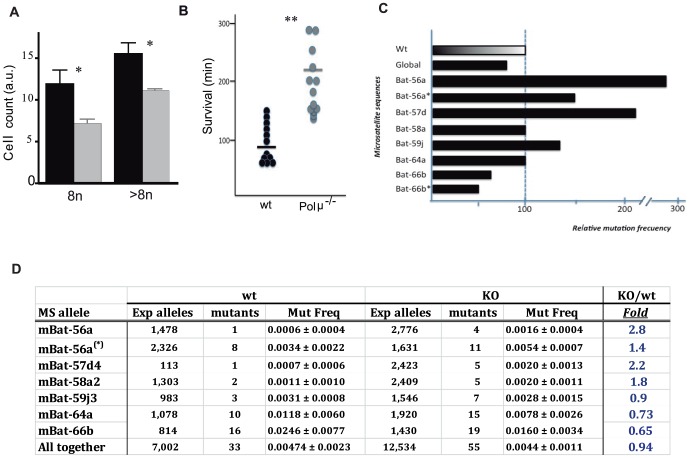
Genetic stability in Polμ^−/−^ mice. (**A**) Polyploidy (determined by PI staining) in hepatocytes from old (14 m) wt (black) and Polμ^−/−^ mice (gray). Data are presented as the relative amounts (arbitrary units) of 8n and ≥8n cells (means ± SEM, 4 samples per genotype. (**B**) Survival (min) of adult (8–12 w) mice after i.v. injection with paraquat (70 mg/kg). Individual data and means are shown for wt (black) and Polμ^−/−^ (gray) mice (n = 13). Data are means ± SEM. (**C, D**) Comparative mutation frequency (MF) in a panel of selected microsatellites (MS) in Polμ^−/−^ mice vs wt animals. The table (**D**) compares individual absolute MF values in the different MS sequences analyzed in Polμ^−/−^ mice relative to wt animals (100%); (*) indicates animals treated with paraquat.

Since Polμ participates in NHEJ, and Polμ^−/−^ liver cells have increased numbers of unrepaired DSB (Figure 1A), we postulate that Polμ deficiency might lead to chromosomal instability. The liver always contains a low and variable percentage of polyploid cells that increases with age and seems to be associated with modifications in cell division fidelity and efficiency, genetic instability or functional decay, or may simply reflect hepatocyte genetic diversity (reviewed by [20]). However, isolated Polμ^−/−^ hepatocytes contained 29% fewer polyploid cells (≥8n; p<0.05) and 40% fewer 8n cells (p = 0.028) than wt samples (Figure 4A). This clearly contrasts with the lower genomic stability defined in BM cells (Fig. S4). These results strongly suggest that Polμ deficiency in mouse liver is linked to a more robust genomic stability (ploidy control) accompanied by downmodulated apoptosis and enhanced preservation of function. Furthermore, Polμ^−/−^ mice were more resistant to acute treatment with paraquat (Figure 4B), a potent inducer of oxidative stress that damages lung, liver, kidney and brain. This response is typically associated with mouse strains with an extended lifespan [21] and thought to be mediated by attenuation of the NAD depletion and reduced redox cycling and oxygen utilization associated with paraquat poisoning [22]; better functionality of Polμ^−/−^ liver could contribute to this increase resistance to paraquat. Analysis of microsatellite (MS) stability in liver cells revealed a mixed picture. We analyzed six mononucleotide MS (mBat-56a, 57d, 58a, 59j, 64a and 66b) that are sensitive instability markers [23]. With no treatment or after moderate treatment with paraquat to amplify putative differences, Polμ^−/−^ and wt mice showed a similar fidelity of repair for mBat-58a and mBat-64a, whereas the mutation frequency of mBat-66b was lower in Polμ^−/−^ mice (48%; p = 0.012), and that of mBat-56a, mBat-57d and mBat-59j was higher (Figure 4C,D). These results indicate that although Polμ deficiency leads to increased global genomic stability in the aged liver, Polμ-dependent NHEJ might contribute to the maintenance of some MS subsets, probably depending on the sequence context.

### Polμ deficiency promotes homologous recombination

**Figure 5:**
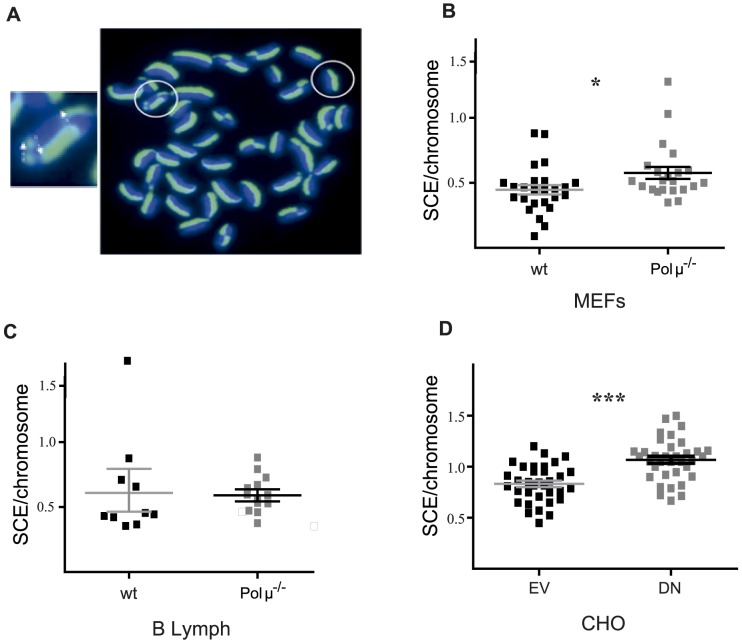
Evaluation of SCE in different cellular models of Polμ deficiency. (**A**) Illustrative example of sister chromatid exchange (SCE) between green chromatids (labeled by BrdU incorporation) and blue chromatids (DAPI stained). The enlarged image to the left shows a chromosome with 3 crossovers (indicated by arrows) in which green and blue are combined in the same chromatid. (**B–D**) Sister chromatid exchange (SCE) in three models of Polμ deficiency: (**B**) MEFs, (**C**) B lymphocytes and (**D**) CHO-KS4 cells expressing DN-Polμ (ΔN); EV = empty vector.

It has been previously reported that deficiency in some core NHEJ factors can be partially compensated by an increase in homologous recombination (HR) repair [3]. To evaluate whether deficiency in Polμ-mediated repair promotes a similar compensatory effect, we analyzed the level of sister chromatid exchange (SCE), an indicator of HR, in several cellular models with reduced Polμ activity Figure 5A–D). Polμ^−/−^ MEFs showed a 29% higher level of SCE per chromosome (Figure 5B), suggesting that the moderately reduced NHEJ in Polμ^−/−^ MEFs [9] [10] leads to a compensatory increase in HR activity. However, this phenomenon is not common to all tissues, since SCE was slightly reduced in total Polμ^−/−^ bone marrow cells (Figure S5A) and unaffected in B lymphocytes (Figure 5C). In addition, preliminary analysis showed that while SCE activity declines with age, there are no significant differences in this trend between Polμ^−/−^ and wt animals (Figure S5A). Analysis of liver (Figure S5B) and bone marrow (unpublished data) by qRT-PCR for HR-related molecules (Rad 50, Rad 51, Rad52 and XRCC2) detected notably higher levels in young Polμ^−/−^ mice, but expression was normal or below normal (70–87%) in old Polμ^−/−^ animals. These results strongly suggest that Polμ^−/−^ liver, which is significantly enriched in G2/M hepatocytes (Figure 1B), also overexpresses key mediators in HR repair.

We additionally studied the effect of expressing a dominant-negative Polμ mutant (DN-Polμ) in CHO-cells. Expression of DN-Polμ (CHO-DN) increased SCE per chromosome by 28.37% (Figure 5D). As a control, CHO-cells were transduced with empty vector (EV) or a retroviral vector for overexpression of wild-type Polμ [24], (unpublished results). We evaluated the impact of Polμ deficiency on direct HR repair by expressing DN-Polμ in the CHO-DRA10 cell line (Figure S5C), which harbors I-SceI substrate constructs that allow direct measurement of the relative amounts of HR and NHEJ events at a defined DSB [25]. Expression of DN-Polμ increased HR repair from 47.7% to 62.3% SCE per chromosome (Figure S5C). These results support the hypothesis that Polμ deficiency triggers a lineage-specific compensatory increase in HR activity with a potential role in the phenotypes described. As mice age, the initial SCE differences are subsumed by the global reduction of SCE activity, suggesting that this modulation of HR is likely not directly related to the improved preservation of function in some organs.

## Discussion

The recent description of delayed brain aging in Polμ^−/−^ mice, associated with reduced error-prone DNA oxidative repair and more efficient mitochondrial function, was the first report of genetic ablation of a DNA-repair function resulting in better maintenance of learning abilities in old mice [11]. This finding was surprising given the moderately increased genetic stability in primary Polμ^−/−^ MEFs and BM cells [9], [10]. Here, our gene expression studies show that liver in old Polμ^−/−^ mice has a modified metabolic profile, with significantly lower-than-normal level of apoptosis (p53-inducible genes) and above-normal genomic stability. The downmodulation of apoptotic processes in old hepatic tissue could contribute, in combination with the lower rate of polyploidy generation, to the delayed liver aging phenotype observed in Polμ^−/−^ mice. Supporting this interpretation, old Polμ^−/−^ mice demonstrated a more robust repair capacity than controls after partial hepatectomy. Other parameters, such as maintenance of subcutaneous adipose tissue and the peripheral ratio of CD4/CD8 cells, also suggested an improved function of those compartments. However, the lack of molecular or electrophysiological signs of delayed aging in Polμ^−/−^ heart indicates that improved preservation of organ function is not generalized throughout the Polμ^−/−^ organism.

Old Polμ^−/−^ mice show lower-than-normal fasting serum levels of total cholesterol, triglycerides and glucose, parameters associated with aging [15]. Polμ^−/−^ animals also consume fewer calories (<10%) than controls (unpublished data), but although this might contribute to the phenotype, these values are far below those used in conventional caloric restriction (CR) regimens (35–46%). Moreover, Polμ^−/−^ mice do not show other features associated with CR (enhanced autophagy, better preserved telomeres, reduction in plasma IGF1 and γH2A-X foci, and reduction of chromosomal alterations) [18]. Finally, the altered aging of Polμ^−/−^ mice seems not to be associated with a lower general susceptibility to cancer.

The significant differences between genetic backgrounds (Figure S2) make it impossible to draw firm conclusions about the impact of Polμ elimination on lifespan. A similar situation emerged recently after analysis of IGF-1 receptor haplo-insufficiency *Igf1r^+/−^* in the C57Bl/6 background, in which no differences in lifespan in males and only a 5% increase in females were found [26], in clear contrast with previous results in 129/J genetic background [27], [28]. This again highlights the determining influence of genetic background on aging phenotypes.

IIS attenuation, telomere attrition, autophagy, oxidative stress and DNA damage repair alterations are among the most established factors influencing aging [29]. Our results show that modulation of telomere attrition and autophagy alterations are not important contributors to the selective preservation of organ function in Polμ^−/−^ mice. GH and IGF-1 levels were non-significantly lower in Polμ^−/−^ mice, and the mild IIS attenuation might be a consequence of the lower accumulation of ROS in aged Polμ^−/−^ animals. A low ROS level is a generally accepted sign of low oxidative stress and therefore low macromolecular and genome damage (reviewed by [30]). Young Polμ^−/−^ mice do not show differences in ROS levels compared with wt animals, but aged Polμ^−/−^ mice have significantly below-normal levels of most ROS species analyzed in BM and thymus (Figure S5D–F), but analysis of macromolecular damage in liver showed that Polμ^−/−^ mice had similar protein damage (carbonyls) and significative increased levels of peroxided lipids (Figure 3B), suggesting a more complex relationship. The longer survival of Polμ^−/−^ mice than wt mice in response to paraquat-induced acute oxidative damage indicates enhanced overall stress resistance. An intriguing interpretation is that low but constant elevation in the basal level of DNA damage in the absence of Polμ during young and adult stages could promote the hormetic development of a more efficient and permanent antioxidant defense and better mitochondrial function during adulthood and old age [31]; this speculation deserves dedicated further research.

Despite the inherent error-proneness of Polμ [4], liver in old Polμ^−/−^ mice shows signs of greater genomic stability (ploidy control), and analysis of overall mutation frequency in a panel of microsatellites (MS) further demonstrated that Polμ^−/−^ mice do not have a globally increased mutation rate, but that the stability of different MS is affected in disparate ways, probably reflecting the sequence context. The results were different when genomic stability was evaluated in BM cells (Figure S4), probably as the result of the quite different proliferative status of both organs.

Overall these results suggest that the effects mediated by Polμ deficiency might be associated with a subset of preferred Polμ target sequences in the genome (as previously demonstrated for VDJ recombination; [6]) that could accumulate variations during aging. The moderate slowing of DSB repair in Polμ-deficient cells [9] would increase the probability of chromosomal rearrangements in highly proliferative organs. The increased rate of HR in some cellular models of reduced Polμ function confirms the high level of cooperation between the main DNA repair pathways (reviewed by [31], [32]) and could mediate some of the positive phenotypes observed in Polμ^−/−^ cells, but in a clear lineage-specific manner. This phenotype has not been previously described for NHEJ deficiency models, probably because the phenotype in those cases is generally much more severe [3].

Our findings suggest a model in which some tissues (brain and liver) in Polμ^−/−^ mice, despite impaired DSB repair capacity under basal conditions, demonstrate a delayed aging phenotype associated with oxidative stress resistance, lower hepatic ploidy and reduced apoptosis. Further research is needed to ascertain the precise mechanisms through which Polμ deficiency promotes functional preservation against aging in these organs.

## Conclusions

Polμ is an accessory error-prone PolX polymerase that contributes to classical NHEJ DNA repair, enabling direct template-dependent synthesis across a DSB, using 3′-protruding ends with no terminal microhomology. Mouse models lacking Polμ (Polμ^−/−^) show altered hematopoiesis homeostasis and DSB repair, and a more pronounced nucleolytic resection of some V(D)J junctions. We demonstrate, however, that despite the reduced DSB repair Polμ^−/−^ mice show an organ-specific enhanced preservation of function with age. Analysis of liver from young and old Polμ^−/−^ animals demonstrated an expression pattern compatible with significant reduction in p53-regulated genes involved in apoptosis; in contrast, the heart showed no differential signature.

Although the best established aging-related regulatory pathways appear not to be affected, Polμ deficiency leads to better genome maintenance in liver, associated with a reduced apoptosis rate and increased resistance to oxidative stress in old mice.

## Supporting Information

Figure S1 **Organ-selective delayed aging in Polμ^−/−^ mice.**
(EPS)

Figure S2 **Comparative lifespan of Polμ^−/−^ mice.**
(EPS)

Figure S3 **Polμ deficiency alters important longevity indicators.**
(EPS)

Figure S4 **Evaluation of bone marrow genetic stability in Polμ^−/−^ mice.**
(EPS)

Figure S5 **Evaluation of SCE in Polμ^−/−^ cells.**
(TIF)

Table S1 **Comparative gene ontology expression analysis in liver of old Polμ ^−/−^ mice.**
(PDF)

Table S2 **Oligonucleotides used for qRT-PCR analysis.**
(PDF)

Text S1 (DOC)
