# Supplementary figures and images for: Polμ Deficiency Increases Resistance to Oxidative Damage and Delays Liver Aging
Source: PLoS One. 2014 Apr 1;9(4):e93074. doi: 10.1371/journal.pone.0093074 (PMC3972199; doi:10.1371/journal.pone.0093074)

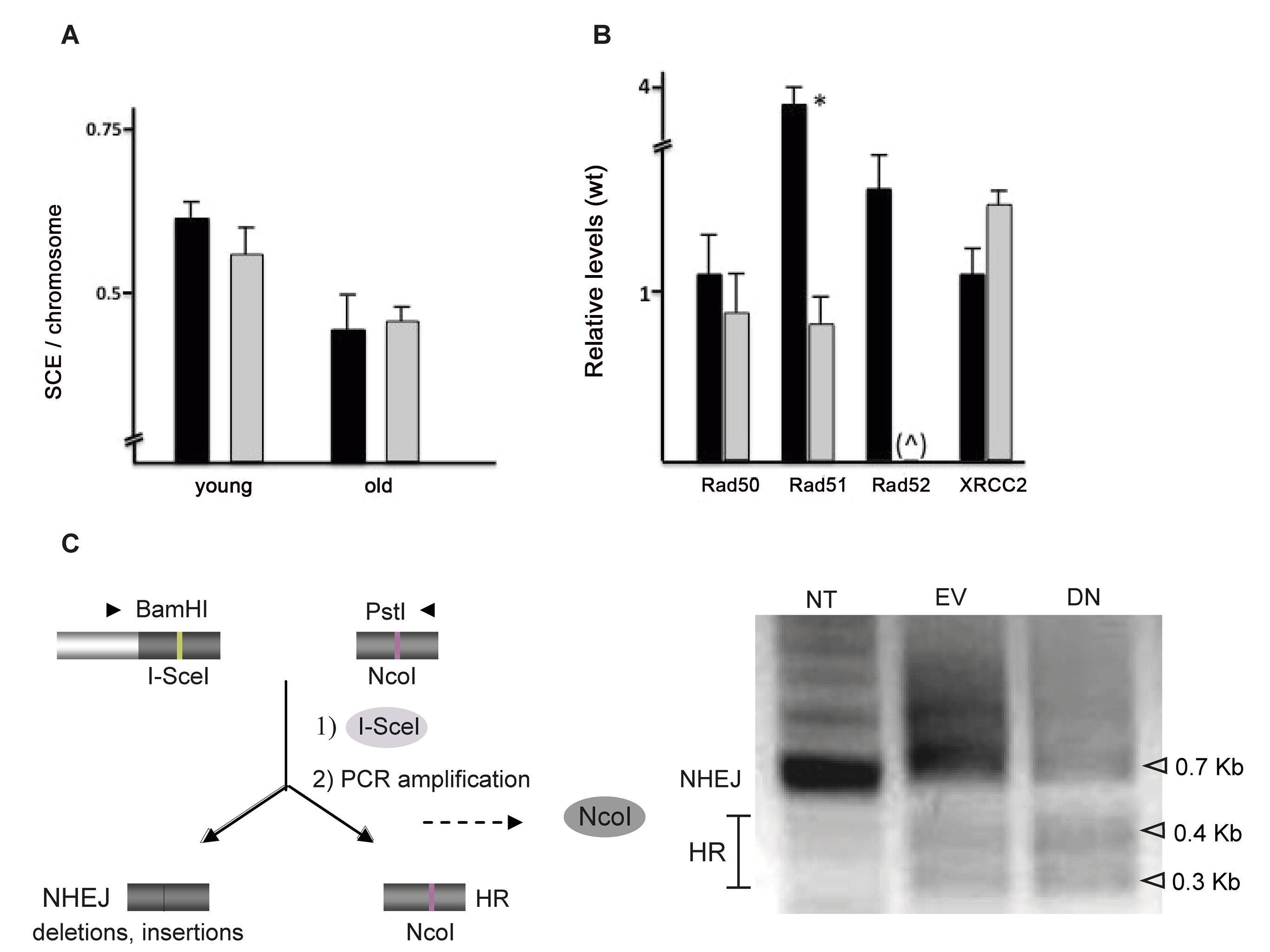

Supplement: Figure S5 — Evaluation of SCE in Polμ−/− cells. (TIF) [file pone.0093074.s005.tif]
