# Supplementary material for: Polμ Deficiency Increases Resistance to Oxidative Damage and Delays Liver Aging
Source: PLoS One. 2014 Apr 1;9(4):e93074. doi: 10.1371/journal.pone.0093074 (PMC3972199; doi:10.1371/journal.pone.0093074)
Supplement: Table S1 — Comparative gene ontology expression analysis in liver of old Polμ −/− mice. (PDF) [file pone.0093074.s006.pdf]

GO term	p value
**Biological processes upregulated in Polμ$^{-/-}$**	
porphyrin metabolism	0,0000000
heme metabolism	0,0000000
pigment metabolism	0,0007000
glutamine family amino acid catabolism	0,0007000
amino acid metabolism	0,0015000
cellular physiological process	0,0015000
secondary metabolism	0,0016000
amino acid catabolism	0,0035000
lipoprotein metabolism	0,0039000
regulation of cholesterol absorption	0,0045000
arginine catabolism	0,0045000
heparan sulfate proteoglycan biosynthesis	0,0045000
amino acid and derivative metabolism	0,0049000
cellular protein metabolism	0,0060000
tRNA aminoacylation for protein translation	0,0068000
tRNA aminoacylation	0,0068000
amino acid activation	0,0068000
cholesterol absorption	0,0074000
lipid digestion	0,0074000
negative regulation of axon extension	0,0074000
negative regulation of axonogenesis	0,0074000
heparan sulfate proteoglycan metabolism	0,0074000
amine metabolism	0,0079000
amine catabolism	0,0082000
nitrogen compound catabolism	0,0082000
cellular macromolecule metabolism	0,0088000
**Biological processes downregulated in Polμ$^{-/-}$**	
antigen presentation	0,0002000
antigen presentation, endogenous antigen	0,0003000
lipid metabolism	0,0006000
antigen presentation, peptide antigen	0,0008000
renin-angiotensin regulation of aldosterone production	0,0012000
angiotensin mediated regulation of renal output	0,0012000
renal response to blood flow during renin-angiotensin regulation	0,0012000
electron transport	
steroid metabolism	0,0012000
generation of precursor metabolites and energy	0,0019000
antigen processing	0,0023000
Golgi vesicle transport	0,0026000
renin-angiotensin regulation of body fluid levels	0,0033000
cellular lipid metabolism	0,0036000
antigen processing, endogenous antigen via MHC class I	0,0037000
regulation of body fluids	0,0043000
localization	0,0044000
establishment of localization	0,0065000
brain renin-angiotensin system	0,0068000
renal blood volume regulation of blood pressure	0,0071000
antigen presentation, exogenous antigen via MHC class II	0,0071000
reproductive organismal physiological process	0,0075000
reproductive physiological process	0,0094000
antigen presentation	0,0094000

Table S1
