# Supplementary material for: Polμ Deficiency Increases Resistance to Oxidative Damage and Delays Liver Aging
Source: PLoS One. 2014 Apr 1;9(4):e93074. doi: 10.1371/journal.pone.0093074 (PMC3972199; doi:10.1371/journal.pone.0093074)
Supplement: Table S2 — Oligonucleotides used for qRT-PCR analysis. (PDF) [file pone.0093074.s007.pdf]

TABLE Supplemental Experimental Procedures

Bid (*BCL-2 Interacting Domain*)		Mm00432073_ml
P21		Mm00432448_ml
Perp		Mm00480750_ml
Acin1 (Acinus; Apoptotic Chromatin Condensator)		Mm00479895_ml
Egln3 (or PDH3, Prolyl-Hydroxylase Domain protein 3)		Mm00472200_ml
Ddit4l/ REDD2 (DNA-Damage-Inducible Transcript 4-like DNA Damage Response *2*).		Mm00513313_ml
Pah	5' CGGCTCCATAGCTGAGAATC 3'<br>5'TCCCGAGACAAGGAAAAGAA 3'	
Knck1	5' CTTTGCCCCCATCTGACA 3'<br>5' TGGAATTGGGACTTCACCTC 3'	
CD209d	5' GTCTGAGAGACCCATCCAGG3'<br>5' GGCCCAACTGGTCATCATAG3'	
Casq1	5' CTCATCAGCAGAAGGCAGGT3'<br>5' TTGACCTGTCAGCTCCACAA3'	
Ddc	5' GGATGTGGTCCCCAGTGTAG3'<br>5' CATGAGAGCTTCTGCCCTTC3'	
eMID2	5' GCTAAGCCGGGTACAGTTCA3'<br>5' CGACCTACCGGGTTTCCTAC3'	
TRAM	5' GACAGGCAGTTCTCAGAGGC3'<br>5' AAACAAACTGAATGAGGCCG3'	
ATG5l	5' ACAGCTTCTGGATGAAAGGC3'<br>5' TGGGACTGCAGAATGACAGA3'	
ATG7l	5' GCCAGGTACTCCTGAGCTGT3'<br>5' GGTCTTACCCTGCTCCATCA3'	
ATG12l	5' CCACAGCCCATTTCTTTGTT3'<br>5' GTCCTCGGCTGCAGTTTC3'	
Map11c3b	5' GCTGCTTCTCCCCCTTGTAT 3'<br>5' CCGAGAAGACCTTCAAGCAG3'	
β-actin	5' GGGGTGTTGAAGGTCTCAAA3'<br>5' AGAAAATCTGGCACCCC3'	

PRIMERS V_H

VH4bS	5´CAGGTGCAGCTACAGCAG3’
JhAS	5´GCTGAGGAGACGGTGACC3’

PRIMERS C_H

C1μS	5´GGACTTCCTTCCCGACTCCAT3’
C2μAS	5´ACGAAGACGCTCACTTTGGGA3’

Junction segments (CHO)

CMV-3	5’-GTACGGTGGGAGGTCTATA3’
CD4-int	5’-GCTGCCCCAGAATCTTCCTCT3’
