# Supplementary material for: Polμ Deficiency Increases Resistance to Oxidative Damage and Delays Liver Aging
Source: PLoS One. 2014 Apr 1;9(4):e93074. doi: 10.1371/journal.pone.0093074 (PMC3972199; doi:10.1371/journal.pone.0093074)
Supplement: Text S1 — (DOC) [file pone.0093074.s008.doc]

***Supporting information***

*Polµ deficiency increases resistance to oxidative damage and delays liver aging*

Beatriz Escudero^1,2^,, Daniel Lucas^2,,&^, Carmen Albo^1^, Suveera Dhup^1^, Jeff W Bacher^3^, Aránzazu Sánchez-Muñoz^4^, Margarita Fernández^4^, José Rivera^5^, Rosa M Carmona^1,2^, Encarnación Fuster^6^, Candelas Carreiro^1^, Raquel Bernad^2^, Manuel A. González^1,2^, Vicente Andrés^5^, Luis Blanco^7^, Enrique Roche^6,8^ Isabel Fabregat^9^, Enrique Samper^1,2,♯^and Antonio Bernad^1,2,♯^

^1^Dpto de Cardiología Regenerativa. Centro Nacional de Investigaciones Cardiovasculares (CNIC). Madrid, Spain. ^2^Departamento de Inmunología y Oncología. Centro Nacional de Biotecnología/CSIC, Campus Universidad Autónoma de Madrid, Madrid, Spain. ^3^Genetic Analysis Group, Promega Corporation, Wisconsin 53711, USA. ^4^Dpto. de Bioquímica y Biología Molecular II, Universidad Complutense, Madrid, Spain. ^5^Departamento de Epidemiología, Aterotrombosis e Imagen. CNIC. ^6^Institute of Bioengineering, Miguel Hernandez University, Elche (Alicante). Spain. ^7^Centro de Biología Molecular Severo Ochoa/CSIC, Cantoblanco, Madrid, Spain. ^8^CIBERobn(CB12/03/30038) Instituto de Salud Carlos. Spain. ^9^Bellvitge Biomedical Research Institut (IDIBELL), L'Hospitalet de Llobregat, Barcelona, Spain.

To whom correspondence should be addressed:

Antonio Bernad

e-mail: [abernad@cnic.es](mailto:abernad@cnic.es)

Footnote 1: **^&^**Present address. Albert Einstein College of Medicine. Michael F. Price Center
1301 Morris Park Avenue, Bronx, NY 10461

Footnote 2: **^♯^**These authors are co-senior

Running title: Polµ deficiency delays liver aging

***Supporting information***

**Supplementary Experimental Procedures**

**Serum analysis.** Blood samples were centrifuged at 3000 rpm for 10 min to obtain sera, which were immediately frozen. All biochemical serum evaluations were performed at the same time to minimize analytical variability, and determined by spectrophotometry with a Selectra Junior Analyzer (Vital Science; reagentes from Spinreact). IGF-1 and GH levels were determined using IGF-1 (Rat/Mouse) or GH (Rat/Mouse) ELISA kits (Demitec). Glucose was measured with a Bayer Contour blood glucose meter and test strips, or a Lifescan OneTouch Ultra glucose meter and test strips.

**Histology.** Young (8-12 weeks) and old mice (18-23 months) were sacrificed and organs were washed with phosphate-buffered saline and fixed in 3.7% formaldehyde overnight at room temperature (RT). Organs were then embedded in 20% sucrose, frozen in OCT and sectioned (8μm) on a cryostat; sections were deposited on poly-L-lysine-coated slides.

**Cell culture.** The different cell types used were cultured in specific medium as detailed below and, when indicated, growth parameters were compared under pro-oxidative conditions (atmospheric, 21% O_2_) or pro-physiological conditions (5% O_2_). Gas proportions were adjusted using tri-gas (N_2_, O_2_, CO_2_) incubators (Termo Urcoa Erloss, Forma Series II Water Jacked Incubator Model 3141).

**CHO (Chinese Hamster Ovary)-derived cell line.** CHO DRA10 cell line [1] was used to measure NHEJ and HR events in the I-Sce-I substrate. Cells were cultured in α-MEM (alpha minimum essential medium, 22561-021) supplemented with 10% FCS, 2 mM L-glutamine, 10 mM HEPES, and 10^5^ U/mL penicillin/streptomycin.

**Mouse embryonic fibroblasts (MEFs).** MEFs were generated from E13.5 day embryos by standard procedures and grown in Dulbecco’s modified Eagle’s medium (DMEM) supplemented with 10% FCS, 2 mM L-glutamine, 10 mM HEPES, 1 mM sodium pyruvate, non-essential amino acids, and 10^5^ U/mL penicillin/streptomycin. Cell were plated, 1x10^6^ cells in a 10-cm tissue culture plate (Falcon), and maintained at 37ºC and 95% humidity in two oxygen atmospheres: 21% O_2_ (standard incubator) or 5% O_2_. Cells were trypsinized and passaged every 3–4 days. Cumulative cell growth was calculated with the formula PD= log(n_f_-n_i_)/log2, where PD is population doubling and n_i_ and n_f_ are the initial and final numbers of cells.

**Activated B lymphocytes.** B lymphocytes were purified from spleen by negative selection with CD43 beads (Miltenyi, 130-049-801) following the manufacturer’s instructions. Cells were cultured in RPMI 1640 (Sigma®, R8758) supplemented with 20% FCS, 2 mM L-glutamine, 10 mM HEPES, 1 mM sodium pyruvate, non-essential amino acids, 0.55 mM 2-mercaptoethanol and 10^5^ U/mL penicillin/streptomycin. Cells were activated by addition of the B-cell mitogens α-CD40 antibody (1 µg/mL; Biosciences®, 16-0402-86) and interleukin 4 (IL-4) (20 ng/ml; R&D Systems).

**Bone marrow (BM) cells.** BM was extracted by flushing the femur with PBS (5mL, aprox.) injected with a G21 needle under sterile conditions. Erythrocytes were lysed by incubating the cell suspension in 0.85% amonium chloride (5 min, 37ºC) and the remaining cells were seeded (2 x 10^6^ cells/mL) in Iscove’s modified Dulbecco medium (IMDM) supplemented with 10% fetal calf serum (Gibco-BRL), 10% conditioned medium from IL-3-producing WEHI-3B cells, and 50 ng/ml human SCF (Stem Cell Technologies).

**Retroviral transduction.** Human Polμ full-length cDNA [2] or the mutant (DN) version was subcloned into the XhoI-EcoRI sites of pLZRI. This vector was derived from the retroviral transfer construct pLZR-CMV-gfp [3] by replacing the EGFP gene with the bicistronic cassette IRES-EGFP from pIRES2-EGFP (Clontech, Palo Alto, CA), preceded by a synthetic multicloning site [4]. The resulting retroviral vectors were named pLZRI-Polμ and pLZRI-PolμDN. High-titer retroviral supernatants (> 5 x 10^6^ infective units/ml) were produced by transient transfection of 293T cells, and CHO cells and MEFs were transduced with these supernatants as described [4]. Transduced (gfp+) cells were fully purified four days post-infection by fluorescence activated cell sorting (FACS) in an EPICS Elite sorter.

**Flow cytometry.** *In vivo* expression of the EGFP retroviral marker was detected by direct observation of cultures with a phase-contrast microscope equipped with a fluorescent lamp and blue filter. FACS analysis was performed on retrovirally-transduced cells in 1 ml PBS (15 x 10^7^ cells/ml). The selective fluorescence threshold was established using the pLZRI-transduced cell population and maintained for other populations. Cell cycle was analyzed by staining, of fixed and permeabilized cells, with 1µg/mL propidium iodide. For the measurement of CD4+ and CD8+ cells in peripheral blood (PB), 50x10^3^ - 50 x10^4^ PB cells were stained for 1h at 4ºC (50μl) with the corresponding primary antibody (CD19 PE, CD3 FITC, CD4 SPRD, or CD8 PE; all from Pharmingen) in PBS plus 1% bovine serum albumin. After 3x washes in 200μl PBS, with centrifugation (5 min, 800xg), samples were resuspended in PBS (500 μl) and analyzed with a Coulter Epics XL-MCL or a Beckman Coulter Cytomics FC 500 cytometer. For FACS staining, 3 ml of diluted primary antibody was used per 10^6^ cells. After washing 3 times in PBS, cells were purified using the Coulter Epics Altra HyperSort System.

**Oxidative stress markers**. Protein carbonyls and thiobarbituric acid reactive substances (TBARS) were determined in perfused liver homogenates as previously described [5], [6], respectively.

**Microarray gene expression profiling**

*Sample labeling and microarray hybridization.* RNA was amplified and labeled using the one-color microarray-based gene expression analysis protocol (Agilent Technologies, Palo Alto, CA, USA). Briefly, 400 ng of total RNA was reverse transcribed using T7 promoter primer and MMLV-RT. cDNA was then converted to aRNA using T7 RNA polymerase, which simultaneously amplifies target material and incorporates cyanine 3-labeled CTP. Cy3 labeled aRNA (1.65 µg) was hybridized to a 4 x 44K Whole Human Genome Microarray (G4112F, Agilent Technologies) for 17 h at 65ºC in 1X GEx Hybridization Buffer HI-RPM in a hybridization oven (G2545A, Agilent Technologies) set to 10 rpm. Arrays were washed according to the manufacturer's instructions, dried by centrifugation, and scanned at 5mm resolution on an Agilent DNA Microarray Scanner (G2565BA, Agilent Technologies) with the default settings for 4x44K format one-color arrays. Scanned images were analyzed with Feature Extraction software (Agilent Technologies).

*Data analysis.* Feature extraction data files were imported into GeneSpring® GX software version 9.0 (Agilent Technologies). Quantile normalization was performed and expression values (log2 transformed) were obtained for each probe. Probes were also flagged (*Present*, *Marginal*, *Absent*) using GeneSpring® default settings. Probes with signal values above the lower percentile (20^th^), and flagged as *Present* or *Marginal* in all replicates in at least one of the two conditions under study, were selected for further analysis. Data were edited and analyzed in R (R Development Core Team) using different packages of the Bioconductor project [7] as well as custom written R routines.

*Data processing.* Data were read into R and processed using the *Agi4x44PreProcess* Bioconductor package as follows. *Agi4x44PreProcess* options were set to use the *MeanSignal* and the *BGMedianSignal* as foreground and background signals, respectively. Data were then background corrected and normalized between arrays using the *half* and *quantile* methods. The *half* method produces a positive background-corrected signal by subtracting the background signal from the foreground signal, while setting intensities below 0.5 to 0.5 to yield positive corrected intensities. Data were normalized between arrays using the *quantile* method [8]. A constant of 50 was added to the intensities before the log transformation in order to reduce the signal variability of low-expressed genes. The AFE image analysis software attaches to each feature a set of flags that identify different quantification properties of the signal. *Agi4x44PreProcess* uses these flags to filter out features that 1) are controls, 2) are out of the dynamic range of the scanner, and 3) are outliers. To keep features within the dynamic range three independent levels of filtering can be applied to ensure that 1) the signal is distinguishable from the background, 2) the signal is found, and 3) the signal is not saturated.

*Statistical analysis.* The differential expression analyses were done using the linear modeling features implemented in the Bioconductor *limma* package. The *limma* package incorporates empirical Bayes methods [9] to obtain moderated statistics.

**Telomere length measurement.** Cell suspensions were washed once in PBS and twice in 0.1% BSA-PBS. Pellets were resuspended (10^6^ cells/mL) in hybridization solution (70% desionized formamide (DF), 20µM Tris pH7.5, 1% BSA, 0.3 µg/mL FITC-labeled telomeric probe (PNA-(CCCTAA)_4_) in mQ water, heated with agitation at 80ºC for 10 min and incubated for 2 h at RT in the dark. Cells were washed twice in 70% DF, 10mM Tris pH7.5-0.1% BSA-0.1% Tween20 -PBS (8 min, 3000 rpm) and once in 10mM Tris pH7.5-0.1% BSA-0.1% Tween20 -PBS (6 min, 2000 rpm). The pellet was resuspended in a solution of 1 µg/mL propidium iodide and 10 µg/mL RNase in PBS, 0.1% BSA and incubated for 2h at RT in the dark. Cells were analyzed in a FACSCanto II cytometer (Becton Dickinson) and telomere length was determined from the relative amount of FITC-signal in live (propidium-iodide-negative) cells.

**Chromosome instability analysis**. After culture of BM cell suspensions for 2 days, 0.1 μM colcemid (Gibco, 15210-040) was added for the last 1.5 h. After swelling in hypotonic buffer (0.56% potasium chloride; 25 min at 37ºC), cells were fixed in methanol/acetic acid (3:1). Translocations in chromosomes 1, 2 and 3 were analyzed in BM (18 months, n=4). Metaphases were fixed in methanol/acetic acid after swelling in hypotonic buffer and spread for hybridization with fluorescently-labeled WCP (whole chromosome paint) probes for chromosomes 1, 2 and 3. The probes were labeled with Cy3, FITC and Cy5 respectively. Images were captured with a Leica DMR microscope fitted with a 10061.3 NA planfluor objective and a Nickon 1200 digital camera. **SKY analysis**. Metaphases were prepared from immortalized cell lines, using a standard cytogenetic protocol for cells fixed in methanol-acetic acid (3:1). Slides were prepared from the fixed material and hybridized using the SKY method according to the manufacturer’s protocol (Applied Spectral Imaging, Migdal Ha’Emek, Israel). Images were acquired with an SD300 Spectra Cube (Applied Spectral Imaging) mounted on a Zeiss Axioplan microscope using the SKY-1 custom-designed optical filter (Chroma Technology, Brattleboro, VT). More than 20 metaphase cells were captured and analyzed for each cell line. Breakpoints corresponding to chromosome abnormalities were assigned based on DAPI banding and the G-banded karyotype of the same cells. A structural chromosome abnormality is considered clonal when observed in two or more cells. Numerical chromosome aberrations are considered clonal when observed in at least three cells. For **Q-FISH**, metaphases were hybridized with a telomeric FITC-labeled PNA-(CCCTAA)_4_ probe (Applied Biosystems) as described [1], with minor modifications. Briefly, post-hybridization washes were performed in PBS, 0.1% Tween 20 at 50ºC (3X 10 min). After image capture for Q-FISH with a Leica DMR microscope fitted with a 100X 1.4 NA planfluor objective and an Olympus DP digital camera, the coverslips were removed with acetone and slides were washed in 2x SSC (5 min). The metaphases were hybridized with Cy3-labeled chromosome 6 painting probe (15μl/slide; Cambio Ltd); 40-50 metaphases were scored for chromosomal aberrations (chromosome and chromatid breaks, dicentrics, rings and Robertsonian-like chromosomes), and 100 metaphases were scored for chromosome translocations (two-color junctions) with the Chr.6 painting probe.

**Supporting Results**

**DNA polymerase μ knockout mice present an organ-dependent delayed aging phenotype**

Old Polμ^-/-^ animals show many features characteristic of delayed aging (Figure S1). Old Polμ^-/-^ animals (18-24 months) showed better preservation of subcutaneous adipose tissue (Figure S1A) and histological analysis also confirmed a similar situation in ovary and pancreas (Figure S1B). In addition, old Polμ^-/-^ mice maintained peripheral blood populations of CD4+ and CD8+ cells at higher levels than wt counterparts (Figure S1C). Old Polμ^-/-^ mice also had lower fasting serum levels of cholesterol (42% less, p= 0.0018), triglycerides (TG; 25% less, p< 0.0001) and glucose (Glu; 32 % less, p< 0.06) (Figure S1D). These results suggest a plausible association with the improved functional preservation phenotype observed in some tissues of Polμ^-/-^ mice. Additionally, elimination of Polμ does not seem to significantly influence tumor susceptibility, since Polμ^-/-^ mice and controls had a similar incidence and distribution of tumors at necropsy (Figure S1E). However, clear differences were detected in the susceptibility to whole body (9 Gy) γ-irradiation. Splenic and testicular cells showed comparable levels of damage, but only Polμ^-/-^ samples presented higher levels of damage in liver, lung, kidney, pancreas and ovary (not shown). These results suggest that Polμ is particularly important under stress conditions that cause DNA damage. Evaluation of thymic lymphomas induced by low-dose γ radiation [10] also revealed overall similar responses in Polμ^-/-^ and wt mice (Figure S1F).

From birth, male and female Polμ^-/-^ mice showed a moderate weight reduction that was maintained throughout life (2-12 months) and a moderately lower-than-normal food intake (<10%; not shown). Although this might contribute to the Polμ^-/-^ phenotype, these values are far below those used in conventional caloric restriction (CR) regimens (35-46%).

Polμ^-/-^ mice in the 129xBALB/c hybrid background, under pathogen-free husbandry conditions, live longer than their wt littermates, housed in parallel (Figure S2A). However, this difference was not confirmed in the C57BL/6 background (Figure S2B) and we therefore cannot conclude any longevity effect directly related to elimination of Polµ. Polμ^-/-^ mice do not present abnormal fertility, in any background (not shown).

**Major pathways contributing to the Polμ^-/-^ phenotype**

Polμ^-/-^ and wt mice also show no significant difference in the circulating levels of IGF-1 at different ages (11-25 months), although levels in Polμ^-/-^ mice tended to be lower with age (10.3-2.5%; Figure S3A). Circulating GH showed a similar pattern (not shown), with a statistically non-significant relative reduction in Polμ^-/-^ mice with age (11.5% at 18 months and 6.1% at and 25 months), relative to wt mice. Low differences were found in glucose tolerance tests in 3-month-old Polµ^-/-^ mice, but these were not maintained in 8-month-old animals (Figure S3B). Therefore altered IIS signaling does not seem to play an important role in the improved function preservation phenotype found in Polμ^-/-^ mice liver. Another main contributor to function preservation is telomere maintenance (reviewed by [11]); however, we found no positive effect of Polµ deficiency on telomere length, either in primary Polµ^-/-^ MEFs (Figure S3C) or in BM or hepatocytes (not shown).

DCFDA staining in thymus (Figure S3E) and spleen (not shown) rendered a similar pattern to BM cells (Figure 3D) with no differences between young wt and Polµ^-/-^ mice but a significant reduction of peroxides in old Polμ^-/-^ mice (25% and 15% lower in thymus and spleen, respectively; p>0.05). However, comparative evaluation of mitochondrial activity in liver did not reveal significant differences between old wt and Polμ^-/-^ mice ([12], unpublished results). In addition, analysis of thymic cells in old Polμ^-/-^ mice (>19 month) in comparison with wt animals demonstrated a substantial reduction in most probes used, affecting most free-radicals analyzed (Figure S3F), including DCFDA (H_2_O_2_, HO., ROO.), DHE (O_2_^-^) and MTG and TMRM (mitochondrial permeability transition pore and energetic state) [13].

We previously reported that Polμ^-/-^ MEFs cultured at atmospheric O_2_ concentration have a higher-than-normal tendency to become senescent [14]. In an attempt to reconcile the data, we further investigated this relationship between oxidative stress and Polμ deficiency, MEFs were cultured at two oxygen atmospheres, 20% and 5% O_2_. Polμ^-/-^ MEFs cultured at 20% O_2_ enter senescence prematurely ([14] and results not shown) but this phenotype was almost completely rescued when the cells were cultured at “pro-physiological” 5% O_2_, accompanied by normalization of chromosome stability (results not shown). As expected [9], premature senescence of Polμ^-/-^ MEFs was accompanied by the upregulation of p19 and, to a lesser extent, of p21 (not shown). In this context we confirmed that relative ROS levels (Polμ^-/-^: wt) are increased at high oxygen tension (20%) and almost identical at low oxygen tension (5%) (Figure S3G). These results suggest that Polμ might be particularly important in stressful environments but less so under regular oxidative stress conditions. The 20% O_2_ condition is likely to be far from physiological, but might reveal the tendency of Polμ^-/-^ cells to be eliminated by senescence when exposed to a sustained stress.

Our results strongly suggest that Polμ deficiency in mouse liver is linked to a more robust genomic stability (ploidy control) accompanied by down-modulated apoptosis and enhanced preservation of functionality. However, in bone marrow (BM), the percentage of aneuploid metaphases was similar in old (17.5-25 months; n=5) Polμ^-/-^ and wt mice (Figure S4A,B), and Polμ^-/-^ BM cells showed a high level of translocations (Figure S4B), in agreement with previous results [14]. Therefore Polμ deficiency does not seem to significantly increase overall genome stability.

**Analysis of the potential role of DNA polymerase μ deficiency in NHEJ/HR equilibrium**

Deficiency in some core NHEJ factors is partially compensated by an increase in DNA repair by homologous recombination (HR) [15]. The modulation of sister chromatid exchange (SCE) activity with age was analyzed in BM, concluding that although SCE activity declines with age, there are no significant differences in this trend between Polμ^-/-^ and wt animals (Figure S5A). Analysis of liver by qRT-PCR for HR-related molecules (Rad 50, Rad 51, Rad52 and XRCC2) detected notably higher levels in young Polμ^-/-^ mice (110-330%, XCRR2 and Rad50, respectively), but expression was normal or below-normal (70-87%) in old Polμ^-/-^ animals (Figure S5B). Levels of Rad52 declined strongly with age, both in Polμ^-/-^ and wt animals (not shown), and only XCRR2 maintained higher levels in Polμ^-/-^ animals at all ages analyzed (112-132%, in young and old animals, respectively). These results strongly suggest that Polμ^-/-^ liver, which is significantly enriched in G2/M hepatocytes (Figure 1B), also overexpresses key mediators of HR repair. We finally evaluated the impact of Polμ deficiency on direct HR repair by expressing DN-Polμ in the CHO-DRA10 cell line (Figure S5C), which harbors I-SceI substrate constructs that allow direct measurement of the relative amounts of HR and NHEJ events in a defined DSB [1]. Although the reaction efficiency was poor, comparison of the intensity of diagnostic DNA bands for HR (0.4 and 0.3 Kb) indicated that expression of DN-Polμ increases HR repair from 47.7% to 62.3% (Figure S5C, right panel). These results support the hypothesis that Polµ deficiency triggers a lineage-specific compensatory increase in HR activity with a potential role in the phenotypes described. As mice age, the initial SCE differences are subsumed by the global reduction of SCE activity, suggesting that this modulation of HR is likely not directly related to the improved preservation of function in some organs.

**SUPPLEMENTAL REFERENCES**

[1] Guirouilh-Barbat J, Huck S, Bertrand P, Pirzio L, Desmaze C, et al. (2004) Impact of the KU80 pathway on NHEJ-induced genome rearrangements in mammalian cells. Mol Cell 14: 611-623.

[2] Ruiz JF, Lucas D, García-Palomero E, Saez AI, González MA, et al.,(2004) Overexpression of human DNA polymerase mu (Pol mu) in a Burkitt's lymphoma cell line affects the somatic hypermutation rate. Nucleic Acids Res 32: 5861-5873.

[3] Yang S, Delgado R, King SR, Woffendin C, Barker CS, et al. (1999) Generation of retroviral vector for clinical studies using transient transfection. Hum Gene Ther 10: 123-132.

[4] Abad JL, Serrano F, San Román AL, Delgado R, Bernad A, González MA (2002) Single-step, multiple retroviral transduction of human T cells. J Gene Med 4: 27-37.

[5] Levine RL, Williams JA, Stadtman ER, Shacter E (1994) Carbonyl assays for

determinations of oxidatively modified proteins. Meth Enzymol 233: 346-357.

[6] Lotito SB, Fraga CG (2000). Catechins delay lipid oxidation and alpha-tocopherol and

beta-carotene depletion following ascorbate depletion in human plasma. Proc Soc Exp Biol

Med 225: 32-38.

[7] Gentleman RC, Carey VJ, Bates DM, Bolstad B, Dettling M, et al. (2004) [Bioconductor:](http://www.ncbi.nlm.nih.gov/pubmed/15461798)

[open software development for computational biology and bioinformatics.](http://www.ncbi.nlm.nih.gov/pubmed/15461798) Genome Biol 5:

R80.

[8] Bolstad BM, Irizarry RA, Astrand M, Speed TP (2003) A comparison of normalization methods for high density oligonucleotide array data based on variance and bias. Bioinformatics 19: 185-193.

[9] Smyth GK (2004) Linear models and empirical bayes methods for assessing differential expression in microarray experiments. Stat Appl Genet Mol Biol 3: Article 3.

[10] Villa-Morales M, Santos J, Pérez-Gómez E, Quintanilla M, Fernández-Piqueras J (2007) A role for the Fas/FasL system in modulating genetic susceptibility to T-cell lymphoblastic lymphomas. Cancer Res 67: 5107-5116.

[11] Parrinello S, Samper E, Krtolica A, Goldstein J, Melov S, Campisi J (2003) Oxygen sensitivity severely limits the replicative lifespan of murine fibroblasts. Nat Cell Biol 5: 741-747.

[12] Lucas D, Delgado-García JM, Escudero B, Albo C, Aza A, et al. (2013) Increased Learning and Brain Long-Term Potentiation in Aged Mice Lacking DNA Polymerase μ. PLOS ONE 8: e53243.

[13] Chen X, Zhong Z, Xu Z, Chen L, Wang Y (2010) [2',7'-Dichlorodihydrofluorescein as a fluorescent probe for reactive oxygen species measurement: Forty years of application and controversy.](http://www.ncbi.nlm.nih.gov/pubmed/20370560) Free Radic Res 44: 587-604.

[14] Lucas D, Escudero B, Ligos JM, Segovia JC, Estrada JC, et al. (2009) Altered hematopoiesis in mice lacking DNA polymerase mu is due to inefficient double-strand break repair. PLoS Genet 5. e1000389.

[15] Li H, Vogel H, Holcomb VB, Gu Y, Hasty P (2007) Deletion of Ku70, Ku80, or both causes early aging without substantially increased cancer. Mol Cell Biol 27: 8205-8214.

**SUPPLEMENTARY FIGURE LEGENDS**

**Figure S1. Organ-selective delayed aging in Polμ-/- mice. (A)** Representative image of hematoxilin/eosin-stained skin sections from wt and Polµ^-/-^ mice (14-18m); subcutaneous adipose tissue is thicker in Polµ^-/-^ mice at all ages studied. **(B)** Representative images of HE-stained tissue preparations of pancreas and ovary from old (18-25m) wt and Polµ^-/-^ mice. Bar indicates 100 μm. In the images relevant structures are indicated: LI, Langerhans islets; F, follicules; aF, antral follicles; CL, corpora lutea and IT, interstitial tissue. **(C)** Percentages of CD4+ and CD8+ cells in peripheral blood from young (3-5 m; n=8) and old (14-25 m; n=10) wt (black) and Polµ^-/-^ mice (gray). Results are means ± SD. **(D)** Fasting levels of total (LDL+HDL) cholesterol (Cho), triglycerides (TG) and glucose (Glu) in serum of young (3-5 m; n=5) and old (14-25 m; n=10-12) wt and Polµ^-/-^ mice. Data are presented as means ± SEM. **(E)** Incidence of spontaneous tumor pathology in organs of old (14-25m; n= 25 for each genotype) wt (black) and Polµ^-/-^ female mice (gray); the chart shows the percentage of animals with tumor pathology in the indicated organs. **(F)** Survival curves of wt (n= 37; black circles) and Polµ^-/-^ animals (n=39; white circles) after induction of thymic lymphomas by repeated low doses of γ-irradiation.

**Figure S2. Comparative lifespan of Polµ^-/-^ mice. (A)** Comparison of percentage survival of female mice from the Polµ**^-/-^** colony (white circles; n= 78) compared with wt mice (black circles; n= 65) in the hybrid 129/BALBc background. **(B)** Survival curves of female mice from the Polµ**^-/-^** colony (white circles; n= 55) compared with wt mice (black circles; n= 55) in the C57Bl/6 background.

**Figure S3. Polµ deficiency alters important longevity indicators. (A)** Age-related IGF-1 serum levels in wt (black; n=9) and Polµ^-/-^ mice (gray; n=11). Each determination was performed in duplicate and data are means ± SEM for each age group: 11 months (wt, n=3, Polµ^-/-^, n=3); 18 months (n=5) and 25 months (wt, n=3, Polµ^-/-^, n=3). **(B)** wt (black; n=10) and Polµ^-/-^ (gray; n=10) mice (3 or 8 months) where challenged with a high dose of glucose (1g/Kg; ip) and monitored at the indicated times for circulating glucose (dg/L). **(C)** Telomere length in MEFs, determined by Q-FISH. Data are means ± SEM (n= 3). **(D, E)** Peroxide levels, determined by flow cytometry and DCFDA-labeling, in wt (black) and Polµ^-/-^ (gray) bone marrow **(D)** and thymus **(E),** from young (4 m) and old mice (>14 m) (n= 8). Data are means ± SEM. **(F)** Levels of free radical species revealed by staining with DCFDA, DHE, MTG and TMRM (see Materials and Methods) in thymic cells from old wt (black) and Polµ^-/-^ mice (gray). **(G)** Evaluation of ROS levels in Polµ^-/-^ MEFs relative to the levels in wt MEFs, cultured at 20% O_2_ (black) and 5% O_2_ (gray).

**Figure S4. Evaluation of bone marrow genetic stability in Polµ^-/-^ mice. (A**) FISH analysis of aneuploidy in chromosomes 1, 2 and 3 in wt and Polµ^-/-^ BM cells obtained from old mice (18-24 months; n=4). The graph shows the percentages of cells with an above-2n (>) or below-2n (<) chromosome number. (**B**) FISH analysis of total aneuploidy and translocations in the populations analyzed in A. Data are presented as the % of the population analyzed (means ± SEM).

**Figure S5. Evaluation of SCE in Polµ^-/-^ cells. (A)** Evaluation of sister chromatid exchange (SCE) activity in bone marrow cells obtained from young (3 months) and old (>20months) Polμ^-/-^ (gray; n=7) or wt mice (black; n=5). Data are means ± SEM. **(B)** qRT-PCR analysis of genes critical for homologous recombination in young (3 months; black; n=5) and old (>20months; gray bars; n=5) Polμ^-/-^ liver; data are expressed as the expression level in Polμ^-/-^ mice relative to wt mice. (^) Indicates that levels are undetectable in old mice in both genotypes. **(C)** Left. Experimental scheme for comparative analysis of HR and NHEJ events in the CHO-derived cell line DRA10 (C10). DRA10 cells, transduced for expression of a dominant-negative mutant of Polμ (DN) or the control empty vector (EV), were transfected with pCMV-I-SceI, harvested 24 h later, and after PCR amplification with the indicated primers (triangles), the resulting products were digested with NcoI and resolved by agarose gel eletrophoresis.

Non-transfected (with the pCMV-I-SceI plasmid) DRA10 cells were used as reference control (NT). Right. DNA bands in DRA10 cells, expressing DN-Polμ (DN), compared with non-transfected cells (NT) and cells transduced with empty vector (EV). Electrophoresis revealed one DNA band of 0.7 kb (NHEJ product) and two diagnostic HR products of 0.4 and 0.3 kb.

**Table S1. Comparative gene ontology expression analysis in liver of old Polμ^-/-^ mice.**

**Table S2. Oligonucleotides used for qRT-PCR analysis.**
